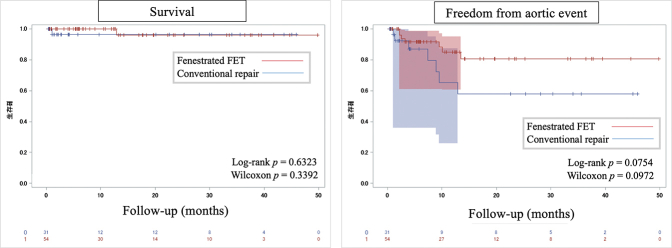# Midterm Results of Fenestrated Frozen Elephant Trunk Technique for Acute Type A Aortic Dissection

**DOI:** 10.1016/j.atssr.2023.12.020

**Published:** 2024-02-01

**Authors:** Takashi Igarashi, Shoichi Takahashi, Hitoshi Yokoyama

**Affiliations:** 1 Department of Cardiovascular Surgery, Fukushima Medical University, Fukushima, Japan; 2 Department of Cardiovascular Surgery, Hoshi General Hospital, Koriyama, Japan

## Abstract

**Background:**

There is limited experience and knowledge of the use of the fenestrated frozen elephant trunk (FET) technique in acute type A aortic dissection (ATAAD). This study’s aims were to assess the clinical outcomes of the fenestrated FET technique for ATAAD and to identify its best practices and pitfalls.

**Methods:**

This study included 101 patients who underwent emergency surgical aortic repair for ATAAD at our hospital between October 2018 and April 2023. We analyzed the perioperative and postoperative outcomes of those treated with the fenestrated FET technique (n = 65).

**Results:**

The rate of postoperative thrombosed false lumen at the distal aortic arch after the fenestrated FET technique was 87%. In most cases, the location of the distal end of the FET was T6 or T7. The rate of distal stent-induced new entry was 8%. The operation time, aorta cross-clamp time, and extracorporeal circulation time were shorter in patients in whom the fenestrated FET technique was performed than in those treated with conventional total arch replacement. Eight patients had secondary aortic events in the follow-up period and were able to be treated by endovascular techniques.

**Conclusions:**

The fenestrated FET technique offers the advantage of low invasiveness during emergent aortic repair. It also provides an option for secondary therapy in cases of late aortic events. Overall, this technique has potential to improve both early and late outcomes of therapy for ATAAD.

**In Short**

▪ We expect the thrombosis rate of the fenestrated frozen elephant trunk (FET) technique to be as high as that of the conventional FET technique.
▪ In most cases, the location of the distal end of the FET was T6 or T7, and the risk of spinal cord ischemia seemed to be low.
▪ The fenestrated FET technique provides surgeons with good exposure of the distal anastomosis and a simple procedure for aortic arch repair.

Acute type A aortic dissection (ATAAD) is a lethal pathologic process that causes aortic rupture, acute heart failure, and organ malperfusion and results in sudden death. In most cases, emergent surgery for aortic repair is needed. Although the early results of emergent surgery have improved in the past few decades, the hospital mortality is still 10% to 20%.^1^^,^^2^

The amount of aorta to be replaced in a patient with ATAAD is an ongoing matter of debate. In recent years, some authors reported their results of comparisons between proximal aortic repair (ascending aorta replacement or hemiarch replacement) and total arch replacement (TAR).^3^ Regarding low invasiveness, limited resection and grafting seem to be better at improving early outcomes in an emergent setting; however, TAR has the potential benefit of reducing late aortic events in the downstream aorta and the reintervention rate.

The fenestrated frozen elephant trunk (FET) technique, which Okamura and colleagues^4^^,^^5^ reported, is a procedure to create a fenestration manually on the FET to fit the bifurcation of supra-arch vessels (SAVs) under direct vision. This technique has some advantages. First, the proximalization of the distal anastomosis of the aortic prosthesis leads to good exposure of the anastomosis and facilitates hemostasis. Second, omitting the reconstruction of some SAVs leads to shortening of the operative duration and low invasiveness. Third, a high rate of positive remodeling of dissected distal aortic arch can be expected, such as in cases of the standard FET procedure.

In this study, we investigated the early outcomes of the fenestrated FET technique for ATAAD, postoperative computed tomography (CT) findings, and secondary aortic events in the follow-up period.

## Patients and Methods

This was a retrospective single-center study, and informed consent for the publication of its data was obtained in the form of opt-out on the website for publication. The institutional review board approved the study (No. R5-10; May 17, 2023).

### Selection of Patients

**Figure 1:**
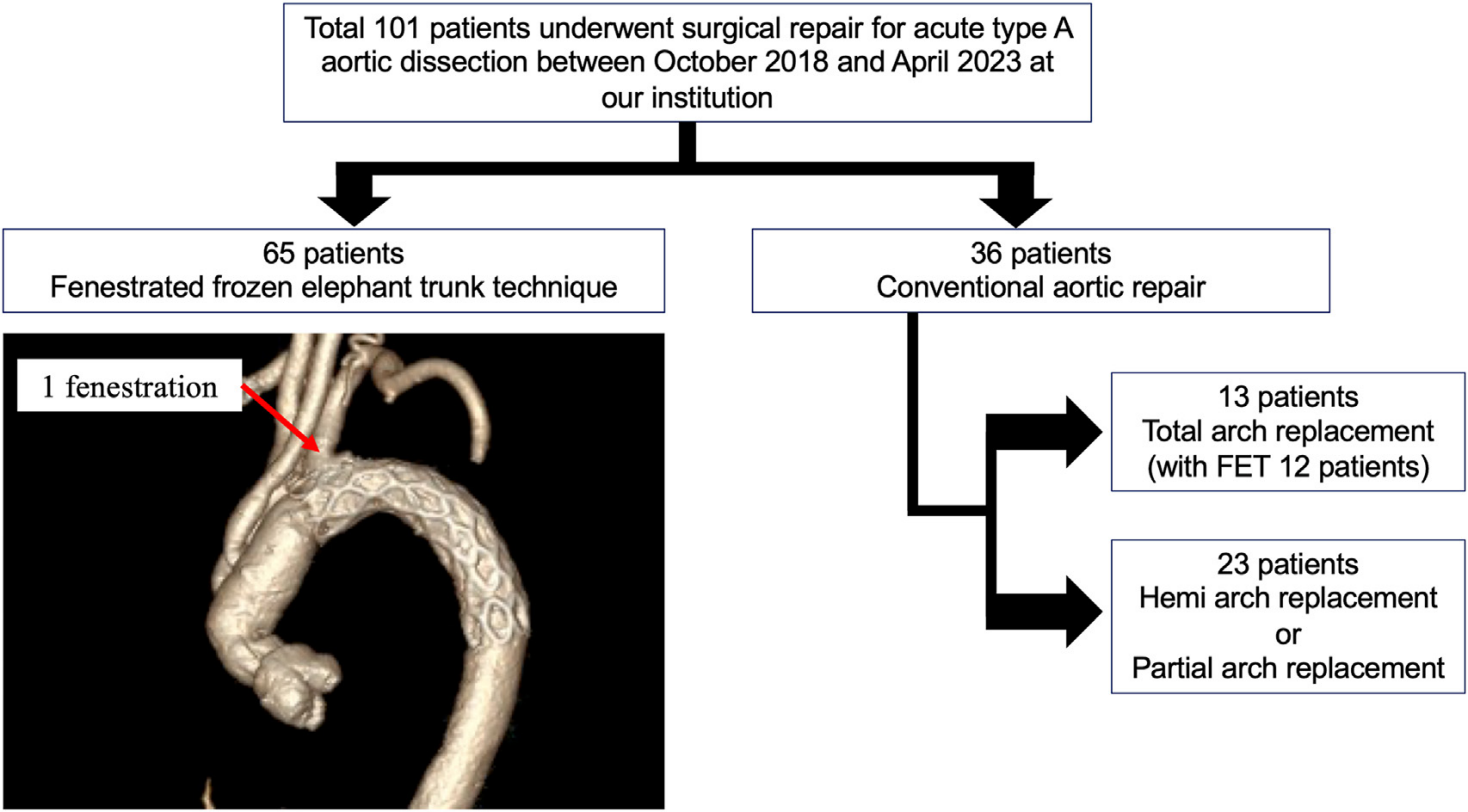
Selection process and number of patients. Between October 2018 and April 2023, 101 patients underwent emergency aortic repair for acute type A aortic dissection at our institution. The fenestrated frozen elephant trunk (FET) technique was performed for 65 patients.

Figure 1 shows the details of selection of the patients. At our hospital, 101 ATAAD patients underwent emergency surgical aortic repair between October 2018 and April 2023. Of these, 65 underwent aortic arch repair with the fenestrated FET technique. The preoperative characteristics of the patients are summarized in Table 1. In cases that had an entry near the bifurcation of the left subclavian artery, conventional TAR was performed because it was difficult to create a fenestration while avoiding a leak of blood from the fenestration toward the entry.

**Table 1:** Preoperative Characteristics of the Patients

Variables	Fenestrated FET (n = 65)
Age, y	63 (57-72)
Male sex	35 (54)
Hypertension	44 (68)
Dyslipidemia	12 (18)
Smoking	36 (55)
CVD	6 (9)
CKD	18 (28)
History of cardiac surgery	3 (5)
Preoperative condition	
Preoperative shock state	8 (12)
Cardiac arrest	2 (3)
Severe AI	7 (11)
Paralysis	5 (8)
Syncope	7 (11)
CT scan findings	
Communicating false lumen in the downstream aorta	50 (77)
Primary entry in ascending aorta	44 (68)
Primary entry in aortic arch	13 (20)
Dissected BCA	38 (58)
Dissected LCCA	20 (31)
Dissected LSCA	12 (18)
Malperfusion	23 (35)
Coronary	2 (3)
Brain	12 (18)
Intestinal	1 (2)
Renal	6 (9)
Spinal cord	1 (2)
Lower extremity	7 (11)

Categorical variables are presented as number (percentage). Continuous variables are presented as median (interquartile range).

AI, aortic insufficiency; BCA, brachiocephalic artery; CKD, chronic kidney disease; CT, computed tomography; CVD, cerebrovascular disease; FET, frozen elephant trunk; LCCA, left common carotid artery; LSCA, left subclavian artery.

### Surgical Procedure

An extracorporeal circulation was established principally by perfusion from the ascending aorta with the Seldinger method. We performed distal anastomosis in an open fashion with circulatory arrest and moderate hypothermia (28 °C at bladder). When the temperature reached 28 °C, circulatory arrest was achieved, and selective cerebral perfusion was established. As an FET, J Graft Frozenix (Japan Lifeline Co, Ltd) was used. During circulatory arrest, the inner diameter of the true lumen of the aortic arch was measured, and an appropriate size was selected for the FET. The length of the FET was decided in a way in which the distal landing of the FET was located within a straightened part of the descending aorta. The fenestration was manually created under direct vision to fit the bifurcation of the SAVs.

## Results

### Operative Data and Early Results

The perioperative data and early results are summarized in Table 2. Resection of the primary tear was achieved in 49 cases (75%). Entry closure with FET was achieved in 9 cases (14%). The number of fenestrations was 1 in 30 cases (46%), 2 in 34 cases (52%), and 3 in 1 case (2%). The length of FET was 60 mm in 3 cases (5%), 90 mm in 27 cases (41%), and 120 mm in 35 cases (54%). As a postoperative neurologic complication, spinal cord ischemia was observed in 1 patient (2%). Recurrent nerve paralysis was not observed.

**Table 2:** Operative Data and Early Results

Variables	Fenestrated FET (n = 65)
Resection of primary entry	49 (75)
Entry closure with FET	9 (14)
No. of fenestrations	
1	30 (46)
2	34 (52)
3	1 (2)
Length of FET	
60 mm	3 (5)
90 mm	27 (41)
120 mm	35 (54)
Concomitant procedure	
CABG	2 (3)
EVT to SAV	6 (9)
AVR	2 (3)
Aortic root replacement	3 (5)
Additional TEVAR	2 (3)
Operation time, min	297 (264-366)
Extracorporeal circulation time, min	190 (175-222)
AoX time, min	114 (103-135)
Early results	
ICU stay, d	4 (2-6)
Hospital stay, d	16 (11-28)
Postoperative neurologic complications	
Stroke	15 (23)
Due to malperfusion	6 (9)
Spinal cord ischemia	1 (2)
Recurrent nerve paralysis	0
Hospital death	8 (12)
Stroke	2 (3)
Rupture of descending aorta	3 (5)
LOS due to coronary malperfusion	1 (2)
Sepsis	1 (2)
Liver dysfunction (liver cirrhosis)	1 (2)
Postoperative CT scan findings	n = 63
Thrombosed false lumen of the aortic arch	55 (87)
dSINE	5 (8)
SAV occlusion	0
Location of distal end of FET (total, n = 63)	
T4	2 (3)
T5	15 (24)
T6	26 (41)
T7	16 (26)
T8	4 (6)
Location of distal end of FET (only FET 120 mm, n = 34)	
T5	2 (6)
T6	18 (53)
T7	13 (38)
T8	1 (3)

Categorical variables are presented as number (percentage). Continuous variables are presented as median (interquartile range).

AoX, aorta cross-clamp time; AVR, aortic valve replacement; CABG, coronary artery bypass grafting; CT, computed tomography; dSINE, distal stent-induced new entry; EVT, endovascular therapy; FET, frozen elephant trunk; ICU, intensive care unit; LOS, low-output syndrome; SAV, supra-arch vessel; TEVAR, thoracic endovascular aortic repair.

**Figure 2:**
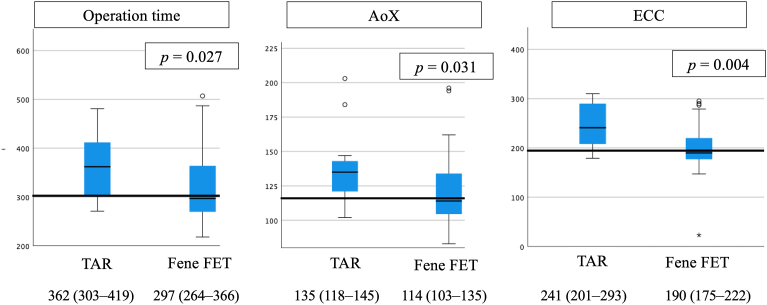
Comparison of the operative time, aorta cross-clamp (AoX) time, and extracorporeal circulation (ECC) time. Between the patients who received the fenestrated frozen elephant trunk (Fene FET) procedure and the patients who received conventional total arch replacement (TAR), there were statistically significant differences in the operation time, aorta cross-clamp time, and extracorporeal circulation time.

During the period of observation, we also performed a conventional TAR in 13 patients. Between the patients treated with the fenestrated FET technique and those treated with conventional TAR, there were statistically significant differences in the operation time (297 [interquartile range, 264-366] minutes vs 362 [303-419] minutes; *P* = .027), aorta cross-clamp time (114 [103-135] minutes vs 135 [118-145] minutes; *P* = .031), and extracorporeal circulation time (190 [175-222] minutes vs 241 [201-293] minutes; *P* = .004; Figure 2).

### Findings of Postoperative CT

Postoperative CT scan was performed in 63 cases (97%). In the postoperative CT scan, a thrombosed false lumen in the aortic arch was observed in 55 patients (87%), distal stent-induced new entry was observed in 5 patients (8%), and no SAV occlusion was observed (Table 2).

The location of the distal end of the FET was T4 in 2 cases (3%), T5 in 15 cases (24%), T6 in 26 cases (41%), T7 in 16 cases (26%), and T8 in 4 cases (6%). In cases of 120-mm FET length (n = 34), the distal end of the FET was T5 in 2 cases (6%), T6 in 18 cases (53%), T7 in 13 cases (38%), and T8 in 1 case (3%).

### Secondary Aortic Event in the Late Follow-up

**Supplementary Figure 1:**
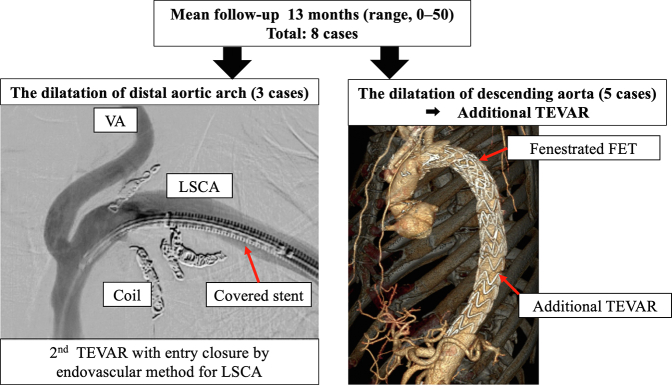


The mean follow-up was 13 months (range, 0-50 months). We observed 8 cases of secondary aortic events. For the 3 cases of dilation of the distal aortic arch, additional thoracic endovascular aortic repair (TEVAR) was performed in 1 case and TEVAR with entry closure by coil embolization and a covered stent for the left subclavian artery was performed in the remaining 2 cases. Additional TEVAR was performed in the 5 cases with dilation of the downstream descending aorta (Supplemental Figure 1).

We compared 2 groups, the fenestrated FET group (n = 54) and the conventional repair group (n = 31; Supplemental Figure 2). Regarding the survival and rates of freedom from aortic events in the late follow-up, there were no significant differences between the groups.

## Comment

In this study, we observed the postoperative thrombosed false lumen at the distal aortic arch in 87% of the patients. Some studies have reported the results of the FET procedure for ATAAD and the rate of postoperative thrombosed false lumen to be 77% to 100%.^6^ Those results are consistent with the results of this study. We expect the thrombosis rate of the fenestrated FET technique to be as high as that of the conventional FET technique.

The deep insertion of the FET beyond the level of the T9 vertebra is known as 1 of the risk factors of postoperative spinal cord ischemia.^7^ In this study, the distal end of the FET was located within T5 to T7 in most cases, and the trend was the same in the patients with 120-mm FET length; postoperative paraplegia was observed in 1 case (2%). From our study, we believe that 120 mm should be the standard choice of FET length in the fenestrated FET technique.

In comparison of the operative duration between the fenestrated FET technique and conventional TAR, the operation time, aorta cross-clamp time, and extracorporeal circulation time were significantly shorter in cases of the fenestrated FET technique than in cases of conventional TAR, which seems to confirm the advantages of the fenestrated FET technique. The fenestrated FET technique provides surgeons with good exposure of the distal anastomosis and a simple procedure for aortic arch repair.

In conclusion, the fenestrated FET technique achieved shorter operative durations than those of the conventional TAR, a rate of postoperative thrombosed false lumen at the aortic arch equivalent to that in cases of the standard FET technique, and the possibility of low invasive endovascular management for the secondary aortic events. Further study is necessary to clarify the long-term outcomes of the fenestrated FET procedure as well as the appropriate indication and advantages of this technique.

## Figures and Tables

**Supplementary Figure 2:**